# The Janus face of Darwinian competition

**DOI:** 10.1038/srep13662

**Published:** 2015-09-10

**Authors:** Arend Hintze, Nathaniel Phillips, Ralph Hertwig

**Affiliations:** 1 Microbiology and Molecular Genetics, East Lansing, MI 48823; 2 BEACON Center for the Study of Evolution in Action, Michigan State University, East Lansing, MI 48823; 3 Department of Psychology, University of Konstanz, Germany; 4 Center for Adaptive Rationality Max Planck Institute for Human Development, Berlin, Germany

## Abstract

Without competition, organisms would not evolve any meaningful physical or cognitive abilities. Competition can thus be understood as the driving force behind Darwinian evolution. But does this imply that more competitive environments necessarily evolve organisms with more sophisticated cognitive abilities than do less competitive environments? Or is there a tipping point at which competition does more harm than good? We examine the evolution of decision strategies among virtual agents performing a repetitive sampling task in three distinct environments. The environments differ in the degree to which the actions of a competitor can affect the fitness of the sampling agent, and in the variance of the sample. Under weak competition, agents evolve decision strategies that sample often and make accurate decisions, which not only improve their own fitness, but are good for the entire population. Under extreme competition, however, the dark side of the Janus face of Darwinian competition emerges: Agents are forced to sacrifice accuracy for speed and are prevented from sampling as often as higher variance in the environment would require. Modest competition is therefore a good driver for the evolution of cognitive abilities and of the population as a whole, whereas too much competition is devastating.

Competion is the basic principle of Darwinian evolution. Over time it brings about better adapted organisms and weeds out weaker physical and cognitive designs. But does this imply that more competition will always result in more adaptive and sophisticated cognition? There are many biological examples of competition driving the evolution of cognitive abilities^1^,^2^,^3^,^4^, and researchers have successfully used competition in genetic algorithms^5^,^6^,^7^. However, there is also evidence that the level of accuracy achieved in human decision making can be lower with competition^8^ than without. In decision making contexts, more competition between agents typically forces them to choose faster. Yet faster responses exact a cost: such responses rely on less information – in the most extreme cases, on little to no information. Less information can (but does not invariably; see Ref. 9) result in a lower level of inferential accuracy. How does evolution trade off accuracy and speed? We show that in less competitive environments accuracy wins over speed, whereas more competition necessitates quicker and less accurate decisions. In extremely competitive situations, agents may rely on minimal information to prevent competitors from choosing first, claiming the most desirable options, and leaving the opponent with an inferior option set. The downside of this strategy of minimal information sampling is the risk of ending up with an inferior option.

Nature provides a good example of this exploration exploitation tradeoff 8,10,11. Hermit crabs habitually outgrow the shell they live in and have to find a new one. When a lone hermit crab finds a new shell, it will investigate it carefully (sample extensively) to make an accurate assessment. If, however, a crab finds a new shell in the presence of a conspecific, the situation is radically different. Hesitance in arriving at a decision can now give a competitor a chance to claim the better shell. Importantly, a hermit crab moving to another shell does not destroy its old one, which can in turn be used by another hermit crab looking for a new shell. When a population of directly competing hermit crabs sorts out inferior shells in this way, it is thus to the benefit of all – a process similar to *crowd sourcing*. This context has been conducive to the evolution of a cognitive strategy that samples sufficiently often. In contrast, imagine a context in which leaving a shell destroys it – or more generally a resource perishes. Here, immediate decisions under direct competition are potentially more successful than is thorough sampling. Such a context could thus prevent organisms from evolving the cognitive ability to explore (sample) thoroughly. It seems that not only the type of competition matters, but also the nature of the resource competed over^12^.

To show how varying degrees of competition can lead to or prevent the evolution of the ability to sample thoroughly, we computationally evolve decision making agents in environments featuring indirect or direct competition. The game we use is a competitive variant of a sampling paradigm^8^,^13^,^14^; a typical example of experience-based decision making^15^. Players sample from an urn, and decide whether it has a higher mean value than an outside reference. The outside reference has a payoff known to the agent, whereas only sampling can reveal the value of the urn. The outside reference could be understood as another urn from which the agent has already sampled exhaustively. Depending on the agent’s decision, the agent will receive either the value of the outside reference or the mean value of the chosen urn. The game is designed in such a way that sampling more frequently from the unknown urn increases the agent’s ability to accurately assess its value (according to the law of large numbers^16^). The accuracy of an assessment given a certain number of samples depends on the underlying distribution. The performance of agents competing in an evolving population will determine their fitness, and players who sample more often will ultimately make more accurate decisions than players who sample little. This holds only as long as competing agents cannot snap up the more desirable urn – that is, when competition is indirect and occurs only on the population level (see Fig. 1A). We call this the *indirect competition environment*.

In the *direct competition environment*, two agents sample from the same urn and try to establish whether it has a higher or lower mean than their own reference urn. As soon as an agent decides either to stay with the reference urn or to pick the sampled urn, the game ends, and the other agent receives the value of its own reference as payoff. If both agents simultaneously pick the same urn, it is allocated randomly to one of them, and the other agent receives the value of its own reference urn as payoff (see Fig. 1B). Therefore, in the direct competition environment, one agent’s decision directly affects the other’s choice environment and, by extension, fitness.

In the third environment, we further amplify the effect of direct competition. Both agents have a reference urn, and are aware of its payoff (outside reference). They can also sample from their opponent’s urn. Agents can stop the game by deciding to keep their urn or choosing to claim the other agent’s urn, leaving the competitor with their own abandoned urn. Now agents not only compete over an outside resource, but can actively decrease their opponent’s fitness (see Fig. 1C). We call this the *extreme competition environment*. We now test how these three environments affect the evolution of sampling strategies and agents’ payoffs.

## Methods

Agents sample from urns that return a value drawn from normal distribution, with a mean of 1, 2, 3, or 4, and a variance of 0.1, 1.0, 3.0, or 5.0, respectively. We distinguish three evolutionary environments, involving indirect competition, direct competition, or extreme competition. In the first and simplest condition, players choose between two urns. The first urn’s mean payoff is known to the player and thus more sampling is not necessary^17^. This urn is called the reference. The second urn’s mean is unknown. The agent can sample from it in order to decide whether to keep the reference or to claim the other urn. Once an agent decides upon an urn it receives the value of that urn as a payoff. In each round, agents choose between three actions: *stay*, *continue*, or *select*. An agent who chooses to *stay* claims the reference urn with the known payoff. An agent who *continues* draws another sample from the unknown urn. An agent who *selects* claims the sampled urn.

To give an example, imagine the direct competition environment, in which two agents are pitted against each other sampling from the same urn. Both agents draw from that urn in the first round. Based on this sample and their own reference urn, each player has to decide between the three options *stay*, *continue*, and *select*. If both players decide to *continue*, they will both draw a new sample from the common urn. In the next round, let us imagine that one player decides to *select*, perhaps because both samples drawn were higher than the reference value, indicating that the common urn has a higher mean than the respective reference urn. The other player decides to *stay*, perhaps because both samples drawn were lower than that players reference. The selecting player now receives the common urn and the staying player keeps its own urn. In a different game, if one player decides to *continue* while the other player either chooses or selects, the game ends, and the player wanting to *continue* retains the reference. If both players decide to *select*, one player is randomly chosen (50/50) to receive the common urn, and the other player retains the reference. A similar conflict can occur in the extreme competition environment, in which both players sample from each others urns. In this environment, if one player decides to *select* while the other player tries to *stay*, it is randomly decided (50/50) whether the players keep their reference urn or receive each others urns.

Each agent can have a range of possible mappings between sampled experiences and actions (stay, continue, or select). The agents’ experiences are represented by two parameters. The first parameter (*m*) is the difference between the average of all samples taken so far and the reference urn. The second parameter (*n*) is the number of samples already taken. Thus, *m* describes how different the two urns are, but this estimate depends on the number of samples taken. A large difference after one sample might be misleading, whereas a large difference after many samples is more likely to be a valid indicator. The parameter *n* allows the agent to take the number of samples into account. To avoid infinitely many samples, we set the maximum number of samples to 100. We always start the game with the continue action so that an agent samples at least once. We encode an agent’s decision strategy in terms of two probabilities that determine the actions it chooses. The first probability is the likelihood of an agent deciding to stay. If an agent decides against staying, the second probability defines the likelihood of an agent continuing or selecting. In order to make the probabilities dependent on the difference (*m*) and the number of samples (*n*), we use a polynomial function that incorporates these two parameters according to the following equation:


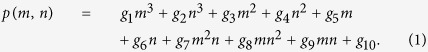


We use this equation to encode each agent’s strategy – once to determine the probability of a player staying or not, and again to determine the probability of a player continuing or selecting. The result of this equation is not limited to be between 0.0 and 1.0. Therefore, a negative value of *p*(*m*, *n*) is defined to be a probability of 0.0, whereas a value of *p*(*m*, *n*) > 1.0 is defined to be a probability of 1.0. To allow for agents to evolve a wide variety of probabilities to stay, continue, or select, we specify two parameter vectors *g*. The first determines the probability of the initial choice (to stay or not). The second determines the probability of continuing or selecting. The values of the parameter vectors *g* can be understood as the genome of the agent and determine its decision strategy. We used a well-mixed population of 1024 agents and, at each update, played each agent against four randomly selected neighbors (when necessary). After each update, 1% of the agents were replaced proportional to the payoff they accumulated over the last updates (Moran death–birth process using roulette wheel selection^18^). Each component of the vectors *g* has a 1% chance to mutate once an agent has been selected to produce offspring. A mutation adds a uniform random number from the interval [−0.5, 0.5] to the mutating component, with no upper or lower limit. The simulation is run for 500,000 updates. At the end of the simulation, a random organism from the population is selected, and the line of descent for this organism is reconstructed. The population usually converges fast to a most recent common ancestor (~15,000 updates, data not shown); we therefore chose the agent at update 450,000 as the representative result of that simulation run. Running the simulation for longer did not change the results, because agents reached the fitness optimum, or could not find ways to further improve their strategies.

Each of the three competitive environments was used in 100 replicate experimental runs. In the indirect competition environment, the agent’s performance depends solely on its own strategy; in the other two environments, performance depends also on the opponent. We therefore measured an agent’s performance by pitting it both against itself and against randomly generated strategies. These randomly generated strategies resemble those strategies created for the first generation. Each representative agent at the end of the simulation competed against itself and against random strategies 1,000,000 times. A new strategy was generated for each of the 1,000,000 games against random strategies.

We considered three outcome criteria: (i) the number of samples taken before selecting an urn, (ii) how well sampling was tuned to the variance in the environment, and (iii) how often each of the urns was taken by that agent. We distinguished between the individual and the population payoff. To this end, the results for 100 representative agents at the end of the simulation are averaged.

## Results

How did the degree of competition affect the decision strategy that evolved, measured in terms of the outcome criteria? Before we turn to the number of samples taken, we first plot the evolved probabilities to stay, continue, or select in Fig. 2. All agents evolved a high probability of staying with their reference urn when the difference between sampled urn and reference urn was negative. This negative difference indicates that the reference urn has a higher payoff and should be preferred. The maximum of this probability changed depending on the intensity of competition. The more competitive the environment was, the higher the likelihood that an agent stayed with the reference urn. Conversely, when the difference between sampled and reference urn was positive, agents evolved a high probability of selecting the sampled urn. Again, the more competitive the environment was, the earlier we find the maximum probability of selecting the sampled urn. Figure 3 shows the number of samples taken before an urn was selected. This number generally decreased with the competitiveness of the environment, and only increased with increasing variance among the samples taken from an urn. This result was expected, as a wider distribution requires more samples to assess the true mean. Interestingly, in the extreme competition environment, the number of samples taken for any variance was just 1. In this environment, agents based their decisions on the minimum sample of 1. Here, strategies evolved to choose extremely fast rather than to gauge the urn’s mean value by drawing more samples.

**Figure 1:**
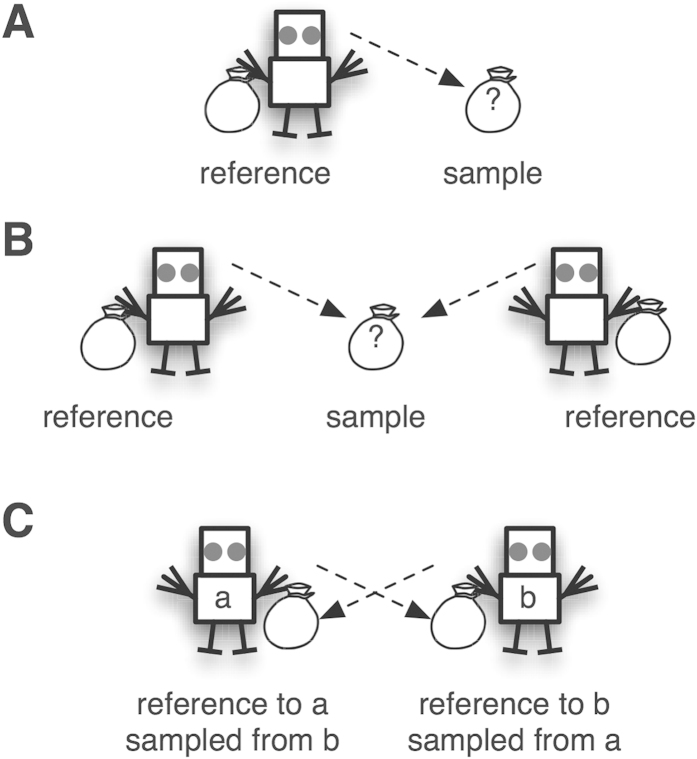
Illustration of the three competitive environments: Panel (**A**) illustrates the indirect competition environment, where the agent, due to sufficient sampling in the past, knows the value of the urn it holds. The agent can sample from the unknown urn and chooses the urn with the higher payoff. Panel (**B**) illustrates the direct competition environment, where both agents compete over the same urn, while knowing the value of their own reference urn. Panel (**C**) illustrates the extreme competition environment, where both agents sample from each other’s urn, and can select the opponent’s urn, leaving their own urn to the opponent.

**Figure 2:**
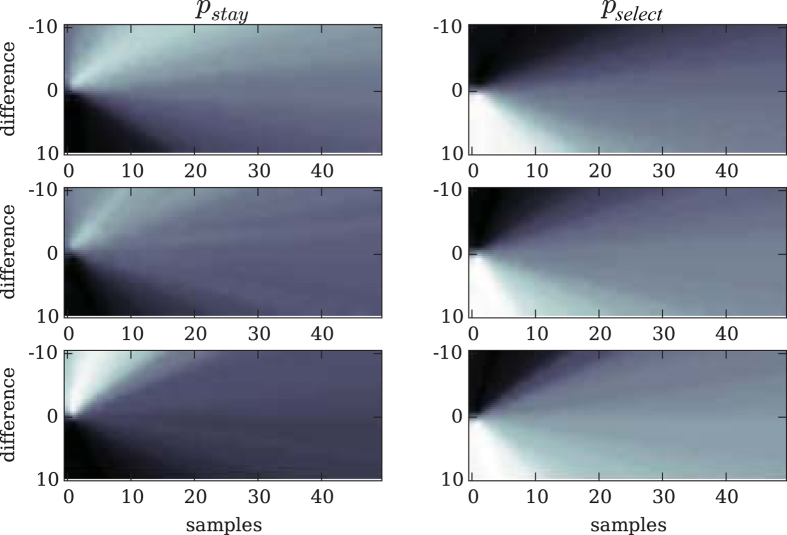
Probabilities of actions: The probabilities to stay (*p*_*stay*_) on the left, and the probability to select (*p*_*select*_) on the right, mapped over the range of differences [−10, 10] between urns (y-axis) and number of samples drawn [0, 50] as gray scales. The probability to continue is implicit since it is 1 − *p*_*select*_. White represents high probabilities, black low. The top panel shows results for indirect competition (see Fig. 1A), the middle panel, results for direct competition (see Fig. 1B), and the bottom panel, results for extreme competition (see Fig. 1C).

**Figure 3:**
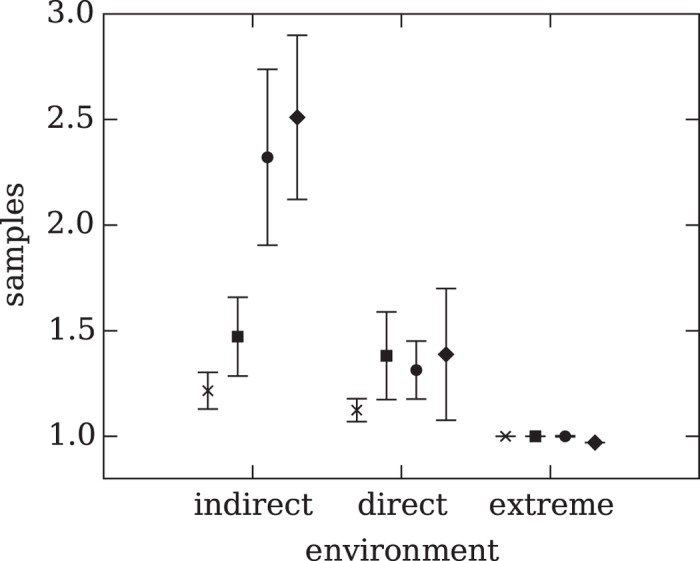
Sampling time: Average number of samples the representative agents took, for each of the three environments: indirect (left), direct (middle), and extreme (right) competition. Each environment was tested using four different variances of the distribution of the urns. Each X indicates a variance of 0.1, squares indicate a variance of 1.0, circles a variance of 3.0, and diamonds a variance of 5.0. The error bars indicate two standard errors.

**Figure 4:**
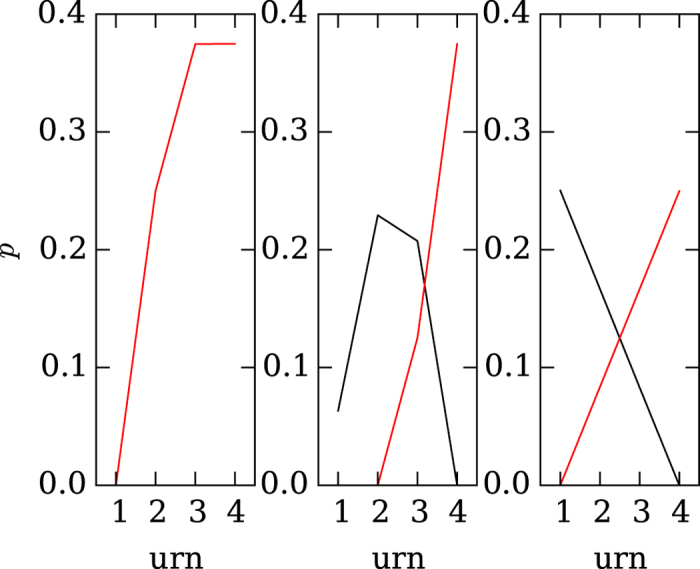
Probability that each evolved strategy playing against itself ended up choosing an urn. The results for the winning strategy are shown in red, and for the losing strategy in black. The left panel shows the probability of a player selecting an urn in the indirect competition environment. Here, each player samples alone, and the probability (*p*) that an evolved strategy decides to select an urn of a given size (x-axis) is shown in red. The middle panel shows the probability of a player selecting an urn in the direct competition environment, and right panel shows the same for players in the extreme competition environment.

The different types of environments affected not only the strategy evolved, but also the payoff. If every agent in the indirect competition environment (see Fig. 1A) plays optimally, the population can be expected to have an optimal gain. An agent in the indirect competition environment can at best always choose the better of the two urns, resulting in a maximum average payoff of 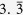. Random choice would result in a payoff of 2.5 (see Supplementary Information). Evolved strategies in this environment come close to this optimal payoff, with 3.12 on average (see left panel of Fig. 4). Here, the individual’s payoff is identical to the average payoff in the population. Furthermore, competition causes agents to evolve strategies that sample dependent on the variance of the environment (see Fig. 3).

In the direct competition environment (see Fig. 1B), two players are confronted with three urns in total. Ideally, the best agent will always choose the best of the three urns, with a payoff of 3.75. The competing agent, if not receiving the highest, but the (remaining) second highest payoff, receives 2.5 (see Supplementary Information for the outcomes of choices).

A perfect strategy playing against itself will win in 50% of cases, and consequently lose in 50% of cases. Therefore, the average maximum payoff is 3.125 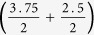. The average observed payoff was 3.01, close to the expected maximum payoff (see the middle panel of Fig. 4). Again, both the individual and the average population payoff was higher than for random choices. However, competition causes agents to evolve only a moderate ability to sample more often in higher variance environments (see Fig. 3).

In the extreme competition environment (See Fig. 1C), both agents compete over two urns, and the losing agent always receives the urn rejected by the player who picked the better urn first. Here, the best possible strategy results in a payoff of 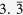; the worst possible strategy in a payoff of just 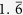; random choice in a payoff of 2.5. Strategies evolved in the extreme competition environment on average received 2.49, that is, the payoff of a randomly ch oosing agent (see right panel of Fig. 4). Here, competition is so extreme that the risk of a competitor beating one to the punch is larger than the benefit gained from drawing one more sample. This prevents the evolution of both repeated sampling and the ability to adjust sampling as a function of environmental variance (see Fig. 3).

**Figure 5:**
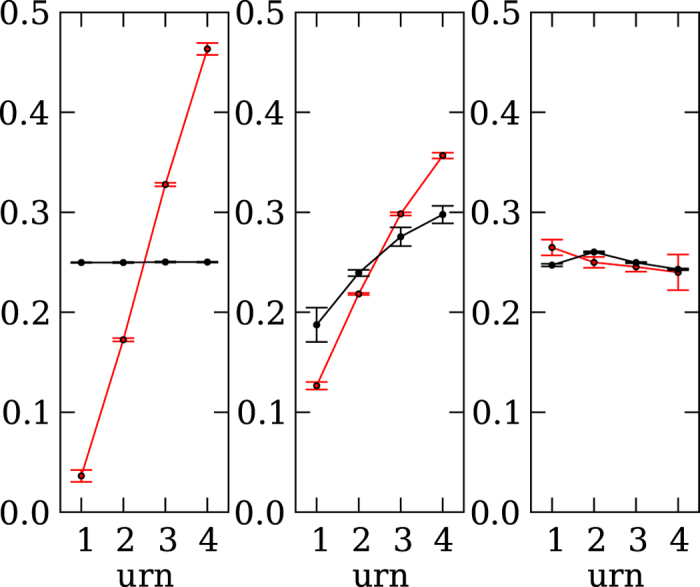
Probability of choosing an urn of a given size in a competition between an evolved strategy playing against random strategies. The left panel shows in red the probability that an evolved strategy in the indirect competition environment will select an urn of a given size (x-axis) and in black the average probabilities for randomly generated strategies. All error bars indicate standard deviations. The middle panel shows the same probabilities for the direct competition environment, and the right panel for the extreme competition environment (red evolved, black randomly generated).

The deleterious effects that extreme competition can have on the evolution of sampling strategies can also be seen when comparing evolved strategies against un-evolved randomly generated strategies. As expected, a random strategy in the indirect competition environment is incapable of choosing the urn with the higher values, whereas evolved strategies are very capable of doing so (see Fig. 5). The same trend can be observed in the direct competitive environment, in which the evolved strategies outperform the random strategies (see middle panel of Fig. 5), but the difference is less pronounced. In the extreme competition environment, evolved strategies make a decision directly after the first sample, which not only prevents the opposing strategy from sampling more, but also results in the evolved strategy making as bad a decision as the random one (see right panel of Fig. 5).

## Discussion

Does more competition always result in better adapted cognition, or might overly competitive environments hamper the evolution of adaptive decision strategies? Organisms evolve in environments of varying competitive pressures, and these conditions shape their behaviors. We designed three environments in which agents evolved decision strategies under indirect, direct, and extreme competition. Indirect competition drove the evolution of repeated sampling and of sampling responsive to the variance in the environment (see Fig. 3). Direct competition led to a similar result, although sampling in this condition was only moderately responsive to the variance in the environment. Extreme competition, in contrast, forced agents to make an immediate decision (based on a single sample), and did not permit the evolution of sensitivity to the variance in the environment. To avoid misunderstandings, we should note that agents evolved optimal decision strategies for the environment they faced. Yet those in the extreme competition environment evolved the least cognitively sophisticated decision strategy, measured in terms of the number of samples and responsiveness of sampling to environmental variance. A related consequence is these agents’ inability to evolve decision strategies that improved not only their own fitness, but also that of each member of the population. In less competitive environments, in contrast, agents evolved a strategy that not only maximized their own payoff, but also allowed the average payoff in the population to increase. Our results show that competition can optimize decision strategies not only to the benefit of the individual, but also to the benefit of the others. There comes a point, however, at which competition becomes too much of a good thing. Under extreme competition, agents evolved behavior that bet exclusively on speed over accuracy. Excessive competition thus reveals the dark side of the Janus face of Darwinian competition: it inhibits the evolution of decision strategies that can adaptively trade off speed against accuracy.

## Additional Information

**How to cite this article**: Hintze, A. *et al*. The Janus face of Darwinian competition. *Sci. Rep*. **5**, 13662; doi: 10.1038/srep13662 (2015).

## Supplementary Material

Supplementary Information
