# Supplementary material for: The Janus face of Darwinian competition
Source: Sci Rep. 2015 Sep 10;5:13662. doi: 10.1038/srep13662 (PMC4564810; doi:10.1038/srep13662)
Supplement: Supplementary Information [file srep13662-s1.pdf]

The Janus face of Darwinian competition

Arend Hintze[1,2,*], Nathaniel Phillips[3], Ralph Hertwig[3]
[1] Microbiology and Molecular Genetics,
[2] BEACON Center for the Study of Evolution in Action,
Michigan State University, East Lansing, MI 48823
[3] Center for Adaptive Rationality
Max Planck Institute for Human Development, Berlin, Germany
* E-mail: hintze@msu.edu

February 9, 2015

Supplementary Information about the outcome of choices

In the indirect competitive environment that agent is randomly presented with two different urns, and thus can experience the following six possible scenarios: 1-2, 1-3, 1-4, 2-3, 2-4, 3-4. Choosing the best in all cases sums to 20, and taking into account that each scenario has the same probability to appear we find a mean payoff for optimal choosing to be $3.\bar{3}$. In case of choosing always the worst, the sum is 10, and consequently the expected least payoff on average is $1.\bar{6}$.

In the direct competitive environment, the two agents are presented with four possible scenarios of payoff in the three urns: 1-2-3, 1-2-4, 1-3-4, 2-3-4. The agent choosing the optimum will receive 15 in total, and because each of the four scenarios occurs with the same probability 3.75 is on average the expected maximum payoff. The second agent could now choose the remaining second highest urn which amounts to 10. The expected best mean payoff for the second agent choosing optimally is therefore 2.5.

In the extreme competitive environment the best choosing agent, similar to the indirect environment can choose the best of two option resulting in $3.\bar{3}$ as the expected average maximal payoff. In turn the opponent would then be left with the lower urn, resulting in $1.\bar{6}$ as the expected payoff. Randomly choosing would result in 2.5 since any of the four options would be chosen with equal probability.
